# Quantib Prostate Compared to an Expert Radiologist for the Diagnosis of Prostate Cancer on mpMRI: A Single-Center Preliminary Study

**DOI:** 10.3390/tomography8040168

**Published:** 2022-08-13

**Authors:** Eliodoro Faiella, Daniele Vertulli, Francesco Esperto, Ermanno Cordelli, Paolo Soda, Rosa Maria Muraca, Lorenzo Paolo Moramarco, Rosario Francesco Grasso, Bruno Beomonte Zobel, Domiziana Santucci

**Affiliations:** 1 Department of Radiology, Campus Bio-Medico University, Via Alvaro del Portillo, 00128 Rome, Italy; 2 Department of Radiology, Sant’Anna Hospital, Via Ravona, San Fermo della Battaglia, 22042 Como, Italy; 3 Department of Urology, Campus Bio-Medico University, Via Alvaro del Portillo, 00128 Rome, Italy; 4 Unit of Computer Systems and Bioinformatics, Department of Engineering, Campus Bio-Medico University, Via Alvaro del Portillo, 00128 Rome, Italy

**Keywords:** prostate cancer (PCa) lesions, multiparametric Magnetic Resonance Imaging (mpMRI), Quantib Prostate software, Artificial Intelligence (AI)

## Abstract

Background: To evaluate the clinical utility of an Artificial Intelligence (AI) radiology solution, Quantib Prostate, for prostate cancer (PCa) lesions detection on multiparametric Magnetic Resonance Images (mpMRI). Methods: Prostate mpMRI exams of 108 patients were retrospectively studied. The diagnostic performance of an expert radiologist (>8 years of experience) and of an inexperienced radiologist aided by Quantib software were compared. Three groups of patients were assessed: patients with positive mpMRI, positive target biopsy, and/or at least one positive random biopsy (group A, 73 patients); patients with positive mpMRI and a negative biopsy (group B, 14 patients), and patients with negative mpMRI who did not undergo biopsy (group-C, 21 patients). Results: In group A, the AI-assisted radiologist found new lesions with positive biopsy correlation, increasing the diagnostic PCa performance when compared with the expert radiologist, reaching an SE of 92.3% and a PPV of 90.1% (vs. 71.7% and 84.4%). In group A, the expert radiologist found 96 lesions on 73 mpMRI exams (17.7% PIRADS3, 56.3% PIRADS4, and 26% PIRADS5). The AI-assisted radiologist found 121 lesions (0.8% PIRADS3, 53.7% PIRADS4, and 45.5% PIRADS5). At biopsy, 33.9% of the lesions were ISUP1, 31.4% were ISUP2, 22% were ISUP3, 10.2% were ISUP4, and 2.5% were ISUP5. In group B, where biopsies were negative, the AI-assisted radiologist excluded three lesions but confirmed all the others. In group-C, the AI-assisted radiologist found 37 new lesions, most of them PIRADS 3, with 32.4% localized in the peripherical zone and 67.6% in the transition zone. Conclusions: Quantib software is a very sensitive tool to use specifically in high-risk patients (high PIRADS and high Gleason score).

## 1. Introduction

Multi-parametric Magnetic Resonance Imaging (mpMRI) is evolving into a main diagnostic imaging for prostate cancer (PCa) [1]. Indeed, mpMRI has been included in the most recent guidelines of the European Association of Urology (EAU) as an important imaging tool to be performed before prostatic biopsy [2], since it improves the detection of clinically significant PCa and decreases the number of unnecessary biopsies [3]. A recent study has shown that mpMRI can improve the detection ratio of clinically significant PCa by 12%, while reducing the risk of insignificant PCa by up to 40% compared to systematic biopsies [4].

However, mpMRI accuracy has been proven to significantly vary between studies [5]. Its usefulness is dependent on the quality of the MRI process, both concerning image quality and reporting. As systematic reporting can improve clinically significant prostate lesions detection and characterization, which are those lesions that may impact the patient’s survival and quality of life, it is recommended to use the Prostate Imaging and Reporting and Data System (PI-RADS v 2.1, American College of Radiology) [6]. Many studies have already reported the high diagnostic performance of this score [7], while inter-reader agreement on PI-RADS categories is moderate, and experience has large effects on specificity of reporting. According to recent research, while an expert and sensitive radiologist would miss 2.6% of clinically significant PCa, a less-sensitive reader missed almost 30% of cancers [8], making inter-reader agreement fair and moderate for higher PI-RADS scores.

The use of computer-assisted software solutions could be a good approach to improve sensitivity in the PCa diagnosis by mpMRI. There are multiple AI radiology solutions on the market. Such solutions could limit the required expertise and inter-reader variability, which is currently present for evaluation of mpMRI data [9,10]. However, there is limited proof for the clinical utility of such AI applications [11].

The aim of this study is to evaluate the clinical utility of an Artificial Intelligence (AI) radiology solution, Quantib Prostate (Quantib B.V. Rotterdam, The Netherlands), by comparing the analysis obtained by an inexperienced first-year radiology resident with the help of the Quantib software and the ground truth set for this study (reports from an expert radiologist and biopsy results) on a series of 108 mpMRI exams.

## 2. Materials and Methods

### 2.1. Patient Population

Our sample is made of 108 mpMRI exams collected from 2019 to 2020 and seen by the same expert radiologist with more than 8 years of experience. When mpMRI was considered as positive, the patient underwent targeted prostatic biopsy.

Of the 108 patients evaluated, 87 underwent prostate biopsy based on the radiologist’s evaluation: 73 (83.9%) prostate biopsies resulted positive, meaning they showed prostate cancer, while the remaining 14 patients did not show prostate cancer (i.e., false positive).

Based on the biopsy results and radiology reports, the mpMRI exams that were followed by a biopsy were divided into groups as shown in Table 1:

### 2.2. MR Imaging

MRI exams were obtained with a 1.5-T system (Magnetom Aera, Siemens, Gurgaon, Haryana, version syngo MR E11) using a pelvic phased-array coil. Patients were examined in the supine position after appropriate preparation.

The prostate gland was studied using axial, coronal, and sagittal T2-weighted turbo spin-echo images using the following setting: Repetition Time (RT) 3520 ms, Echo Time (ET) 114 ms, Field of View (FOV) 200 mm, slice 30, slice thickness 3 mm, and distant factor 10%. T1-weighted fast spin-echo transverse images were then acquired with the following parameters: TR 426 ms, TE 11 ms, FOV 330 mm, slice 30, slice thickness 3 mm, and distant factor 10%. The imaging protocol included diffusion images with b-value of 500–1000–1500–2000 s/mm^2^ and Apparent Diffusion Coefficient (ADC) maps.

The last series performed was a 3D Volumetric interpolated breath-hold examination (VIBE) T1-weighted fat sat in axial plane (TR, 4.3 ms, TE 1.62 ms, FOV 260), immediately following intravenous bolus injection of 0.15 mL/kg body-weight dose of paramagnetic contrast medium (gadoteric acid 0.5 mmol/mL) at rate of 3 mL/s.

A written informed consent was obtained from each patient who performed the mpMRI.

### 2.3. Pathological Validation

Pathological comparison was made in all the patients of groups A and B. After the mpMRI exam, the patients underwent a trans-rectal prostate biopsy, as is the current standard. The biopsies were conducted both as MRI/Utrasound (US) real-time fusion biopsy at targeted sites (Urostation Koelis, Grenoble, France) and as systematic-14 cores-random biopsy on the whole prostate parenchyma. The 14-cores scheme was applied for all patients and included the sampling of apex, basis, equatorial zone, and right and left of the prostatic gland tissue.

We used the International Society of Urological Pathology (ISUP) grading of prostate cancer for the pathological correlation, reporting as five groups, ISUP grades 1–5, respectively, for Gleason scores ≤ 6, 3 + 4 = 7, 4 + 3 = 7, 8, and 9–10 [12].

As already said in the Introduction, each lesion seen by the radiologist was reported using PI-RADS v. 2.1 scores 1–5, where scores 1 and 2 correspond to benign tissue, score 3 shows inconclusive results, and scores 4 and 5 suggest malignant tissue.

### 2.4. Quantib Prostate Software

In this study, the inexperienced radiologist was assisted by Quantib Prostate (Quantib B.V., Rotterdam, The Netherlands), which we denote AI-assisted radiologist. The software aims to improve the radiology workflow of prostate diagnostic MRI. Quantib Prostate offers features for the reading of prostate MRI in one workflow. The semi-automated combination of bi-parametric data provided supports Region of Interest (ROI) determination and enables prostate lesion evaluation. It allows calculation of lesion volume through its AI-bolstered segmentation. Furthermore, a report including Prostatic Specific Antigen (PSA) density, quantified volumes, PI-RADS v2.1 scoring and key images of suspicious regions is automatically generated. The current version 1.3 is CE marked and cleared by the Food and Drug Administration (FDA).

The Quantib Prostate workflow consists of three steps:

1. PSA density analysis, with the automatic segmentation of the prostate, which can be reviewed and modified by the radiologist;
2. The multiparametric MRI analysis, where standardized assessment of MRI includes the ability to add, edit, and inspect ROIs and score them according to PI-RADS general scoring, finding, reviewing, and approving the results;
3. Results are exported and a standardized report is created.

Imaging output includes prostate contours and segmentation, ROI contours and segmentation, and secondary capture series of an image-on-image overlay, which can be the bi-parametric combination image.

**Figure 1:**
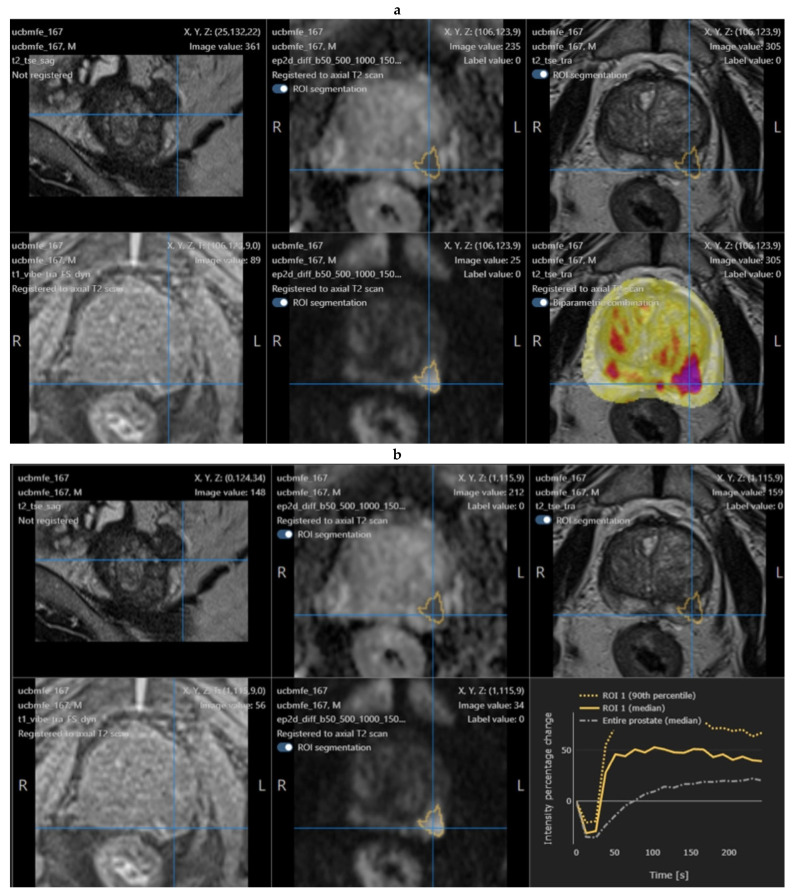
Example of a positive mpMRI prostate nodule, showing the original software interface with the segmentation of the lesion (**a**) and post-contrast enhancement curve (**b**).

An example is shown in Figure 1.

### 2.5. Study Design and Statistical Analysis

All mpMRI exams were evaluated in the clinical workflow by one experienced radiologist. This evaluation was done in the clinical routine, thus, before biopsy and, therefore, blinded to biopsy results. This was done on a dedicated workstation (Syngo.via, Siemens, Erlangen, Germany).

The inexperienced radiologist re-evaluated all cases blinded to the diagnosis and biopsy results.

Both the expert radiologist and the inexperienced radiologist helped by Quantib analyzed all the mpMRI exams reporting for each lesion:

- Prostatic zones: transition zone (TZ) or peripherical zone (PZ);
- Side and localization: right or left; apex, equatorial, or base;
- Largest axial dimension;
- Lesion volume (calculated by Quantib);
- PI-RADS.

The diagnostic capacity of an expert radiologist (more than 8 years) was compared with the diagnostic capacity of an inexperienced radiologist, first-year resident (the AI-assisted radiologist).

The evaluations were conducted as lesion analysis, while a “per patient” analysis was excluded as there could be up to three lesions per patient. Biopsy reports were compared to the ROIs identified by the experienced radiologist as part of the current clinical workflow, as well as to the ROIs indicated by the AI-assisted radiologist. True positives (defined as correspondence of malignancy between lesion identified by radiologists and target biopsy), false negatives (defined as positive result at random biopsy without identification by the radiologist), and false positives (defined as negative result at target biopsy with positive identification by the radiologist) could, thus, be assessed for all patients who underwent biopsy.

The statistical analysis for the prediction of sensitivity and positive predictive value (PPV) was performed by SPSS v. 22 (IBM, Armonk, NY, USA) using traditional computational analysis. We calculated sensitivity as number of true positives/(number of true positives + number of false positives) and PPV as number of true negatives/(number of true negatives + number of false positives).

One-sided Student’s *t*-test was used to compare age, PSA levels, prostate volume, lesion volume, and lesion axial dimension between the three groups of patients. The statistical significance was set at 0.05.

## 3. Results

The clinical information (age, PSA, and the prostatic volume) and the MRI prostatic lesions details (largest axial diameter and volume) for each group are reported in Table 2 and Table 3, respectively.

In group A, the expert radiologist found 96 lesions in 73 mpMRI exams; of them, 17.7% were PIRADS 3, 56.3% were PIRADS 4, and 26% were PIRADS 5. The AI-assisted radiologist found 121 lesions; of them, 0.8% were PIRADS 3, 53.7% were PIRADS 4, and 45.5% were PIRADS 5. At biopsy, 33.9% of the lesions were ISUP 1, 31.4% were ISUP 2, 22% were ISUP 3, 10.2% were ISUP 4, and 2.5% were ISUP 5.

In Table 4, the lesions found by the expert and the AI-assisted radiologists are shown in relation to their nature (location, ISUP, and PIRADS classification).

**Figure 2:**
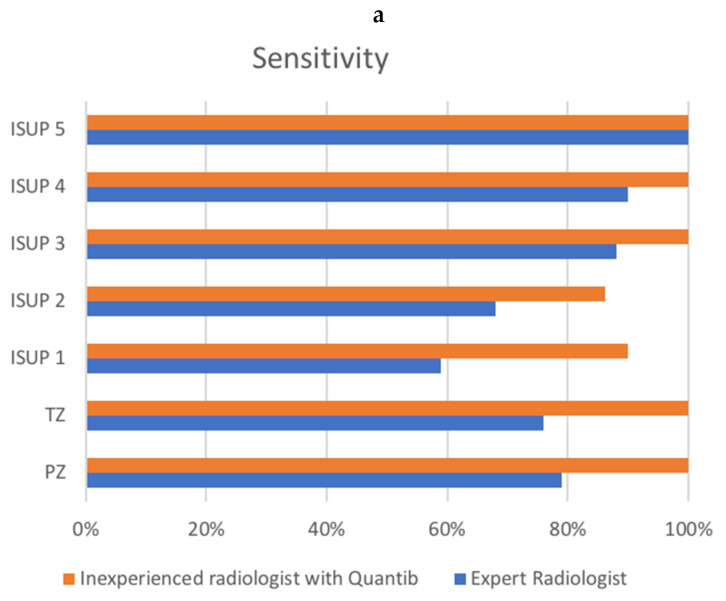

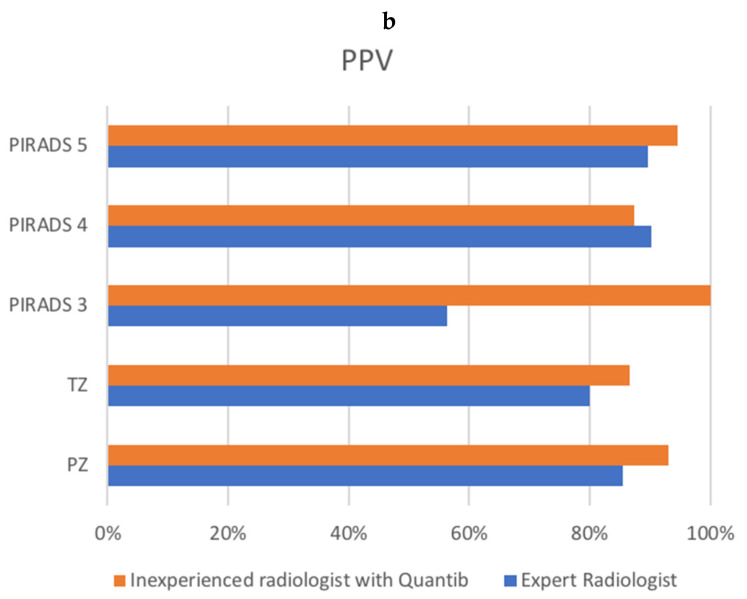
Comparison of sensitivity (**a**) and PPV (**b**) between the radiologist and the AI-assisted inexperienced radiologist, for groups A and B combined. *X*-axis represents percentages. (**a**) shows ISUP on the *y*-axis, as well as transition zone (TZ) and peripheral zone (PZ) sensitivity. (**b**) shows PI-RADS v2.1 score, as well as transition zone (TZ) and peripheral zone (PZ) positive predictive value (PPV).

Evaluating group A, the expert radiologist reached a sensitivity of 71.7 and a PPV of 84.4%, while the AI-assisted radiologist reached a sensitivity of 92.3% and a PPV of 90.1%. Figure 2 shows the sensitivity and PPV for groups A and B combined.

Analyzing the cases which resulted in false negatives from expert radiologist evaluation and true positives from AI-assisted radiologist analysis (23 ROIs), 12 were ISUP 1, 7 were ISUP 2, 3 were ISUP 3, and only 1 was ISUP 4.

In group A, nine cases were false negatives for both the expert radiologist and AI-assisted radiologist (four ISUP 1 and five ISUP 2).

In group B, the expert radiologist found 17 lesions in 14 mpMRI exams (47.1% PIRADS 3 and 52.9% PIRADS 4). The AI-assisted radiologist found 14 lesions in the same 14 mpMRI exams (71.4% PIRADS 3 and 28.6% PIRADS 4). Moreover, 21.4% were in the PZ and 78.6% in the TZ.

In group C, the expert radiologist did not find any lesions. The AI-assisted radiologist found 37 lesions in 21 patients; of them, 86.5% were PIRADS 3, and the rest were PIRADS 4; moreover, 32.4% were in the PZ and 67.6% in the TZ.

## 4. Discussion

In this study, we demonstrated that AI software solutions for mpMRI can increase sensitivity and PPV in PCa diagnosis. Specifically, we observed a sensitivity of 92.3% for patients with positive mpMRI and positive biopsy (Group A).

It is commonly accepted that the use of AI software solutions can complement the diagnostic performance of the radiologist for PCa [13]. Greer et al. [14] studied a CAD assistance and observed an increase in sensitivity (98% vs. 92.7%), particularly in the PZ. Differently than in our study, they mostly used prostatectomy patients, who can have clearer mpMRI-detectable lesions than men with just an elevated PSA across risk stratification. Cameron et al. [15] proposed a quantitative feature model, MAPS (morphology, asymmetry, physiology, and size), based on radiomics, and reported a sensitivity of 86%. Khalvati et al. [16] proposed a MPCaD (multiscale radiomics-driven frame-work) for PCa, obtaining a sensitivity of 82%.

Considering only lesions with ISUP 4 or 5, our results showed that an inexperienced AI-assisted radiologist reached a high sensitivity, which is equal to or possibly slightly higher than the experienced radiologist. ISUP classification is the current gold standard for prognostication of PCa [17]. ISUP scores are used for stratification of patients into different risk groups and might predict Gleason scores in vivo [8].

Concerning PI-RADS scores, our results indicated that the AI-assisted radiologist had a similar or better PPV for the PIRADS scoring. This is probably due to the mainly higher PIRADS lesions, where the inexperienced radiologist gave a PI-RADS 5 to almost double the number of lesions compared to the experienced radiologist.

Recently, some studies have verified the possibility to use radiomics to attribute PI-RADS scores. Wang et al. [18] used a support vector machine (SVM) based on a radial-basis-function (RBF) kernel to analyze radiomic features extracted from MRI sequences, and they reported that radiomic features can improve the diagnostic performance (from 79% to 94.4% in the PZ and from 73.4% to 91.6% in the TZ). In this study, because of the lack of an MR/TRUS fusion-guided in-bore biopsy, a histological/radiological correlation was established through a systematic, consensus-seeking correlative review of the histological and MR findings by a genitourinary pathologist and a radiologist, without a reproducible method.

Hou et al. [19] developed a model integrating data extracted from the most important MRI sequences in improving diagnostic accuracy in PI-RADS 3, which differentiated clinically significant PCa from indolent and normal cases.

As seen before, the high heterogeneity of studies, mostly due to the different patient samples, software characteristics, and settings that are used, makes a real comparison between each software difficult.

In our study, we reported the diagnostic performance of an expert radiologist and of an inexperienced AI-assisted radiologist in a setting of different ISUP and PIRADS, without considering the radiomics of each lesion. Other studies are needed for that.

In this set, the false negatives identified by the expert radiologist were 23, of which 82.61% were of ISUP class 1 or 2, while the false negatives detected by the AI-assisted radiologist were 9, and of these 100% were of ISUP class 1 or 2. This means that the diagnostic accuracy of the AI-assisted radiologist is higher than the expert radiologist, when considering sensitivity and positive predictive value for the more aggressive lesions, but these results also indicate that in the unidentified cases the tumor lesions showed very low aggressiveness (ISUP 1 or 2).

Group B was set in order to assess the capability of the AI-assisted radiologist to exclude false positives, and group C was set in order to evaluate the AI-assisted radiologist on cases that were identified as negative by the expert radiologist. In group B, the false positives detected by the expert radiologist were 17, while those detected by the AI-assisted radiologist were 14. Furthermore, 53% of the lesions identified by the expert radiologist had been stratified as PIRADS 4, while only 28% of these lesions identified by the AI-assisted radiologist had this score.

In most cases, the AI-assisted radiologist showed PIRADS 3 lesions that were actually negative at all biopsy locations. Thus, while there was a strong agreement on the identification of lesions between the expert radiologist and the inexperienced radiologist with Quantib software, the diagnostic accuracy did not significantly increase. In group C, the AI-assisted radiologist found new lesions which are likely to be false positives. Both in group B and in group C, the majority of lesions were in the TZ (67.6–78.6%). One possible explanation for the high rates of false positive in groups B and C is that the Quantib software did not detect a main lesion, but it did detect many mild suspected areas in the TZ and less frequently in the PZ. In these cases, benign prostatic hyperplasia nodules could be mistaken for PCa lesions. Indeed, the prostate volume was significantly larger in groups B and C (Table 2), which is negatively correlated to PCa and could corroborate this hypothesis.

In some cases, we observed a suspected area on mpMRI, with a positive biopsy in more than two cores that correspond to that area; so, to calculate sensitivity and PPV, we considered only the higher Gleason score (GS) between the cores corresponding to the lesion. Therefore, our percentages can be slightly different considering the ISUP stratification or lesion stratification. For the group of patients with no biopsies performed (group C), a direct comparison between the radiologist’s report and the AI-assisted radiologist was performed by comparing the number of ROIs. As our clinical practice does not perform biopsies on patients with negative mpMRI, the number of true negatives or false negatives cannot be calculated.

However, since this version of the Quantib software mostly uses DWI to consider an area at risk of PCa, considering that the most important MRI sequence for the TZ evaluation is the T2-weighted scan, it is probable that Quantib over-considered some areas in the TZ as at risk. Based on communication of our results to Quantib, their next software version (already available) includes technical improvements that could possibly reduce the number of false positives in the transitional and central zone, by giving greater importance to T2-weighted imaging features of PI-RADS 3,4, and 5 lesions in the PZ and the TZ.

There are several obvious limitations to this study. This is a single-center, single-reader, and single-software study. Even more, no nodules with PIRADS score 1 or 2 were included in the study. This study should, thus, be extended, replicated, and performed with multiple software packages in order to be generalizable. Furthermore, as negative cases (group C) did not undergo biopsy, and follow-up of these men was not available for this study, the specificity and negative predictive value could not be assessed. As a future study perspective, we would consider also subjecting the PIRADS 1 and 2 nodules and deepening the learning curve of an inexperienced radiologist, by evaluating their diagnostic performance before and after using the software. Our next studies will include a post-hoc analysis to evaluate patients’ follow-up (PSA and other anamnestic information, for example) and analyze falsely identified and falsely missed cases in order to investigate their classification.

## 5. Conclusions

The role of mpMRI in the management of prostate cancer lesions has been well-established. MRI allows the correct identification of PCa lesions, which is fundamental both to guide the execution of targeted biopsies and for the lesions contouring in case of radiotherapy treatment. However, the interpretation of a prostatic MRI is not always performed by dedicated radiologists. In our study, Quantib Prostate software was proven to be a very accurate tool for the initial diagnosis of PCa on mpMRI, and we demonstrated that it could provide a supporting tool even to untrained radiologists.

## Figures and Tables

**Table 1:** Patients grouping.

Group	A	B	C
Patients	73 (67.6%)	14 (13%)	21 (19.4%)
Notes	positive mpMRI positive biopsy (positive target ± positive random biopsies)	positive mpMRI negative biopsy	negative mpMRI no biopsy confirmation

**Table 2:** Age, PSA, and prostate volume distribution in the three groups of patients. Prostate volume is significantly (* *p* < 0.01) different between group A and group B.

	Group A (n = 73)	Group B (n = 14)	*p*-Value	Group C (n = 21)
Age (years)	67.7 (52.4–84)	66.5 (54.9–75.2)	0.8	64.3 (56.2–73)
PSA (ng/mL)	8.2 (2.7–25)	7.6 (3–13.2)	0.74	6.3 (1.8–9.2)
Prostate volume (mL)	56.6 (21–137.9)	93.3 (44.4–182.7)	<0.01 *	81.1 (28.7–157)

**Table 3:** Mean, median value, and range of volume and largest axial diameter of lesions in groups A, B, and C, according to the Quantib software.

	Group A	Group B	*p* Value	Group C
Lesion Volume (mL) Mean, median (range)	0.71; 0.56 (0.06–3.69)	0.65; 0.5 (0.02–2.06)	0.72	0.24; 0.2 (0.04–0.62)
Largest axial diameter (mm) Mean, median (range)	14.8; 14.4 (4.6–40.9)	13.2; 12.8 (3.9–26.7)	0.81	10.1; 9.9 (5.2–16.6)

**Table 4:** Comparison between radiologist and Quantib about sensitivity and PPV, considering location, Gleason score, and PIRADS of lesions. (PZ: peripheral zone; TZ: transitional zone).

	Sensitivity		PPV	
	Radiologist	Quantib	Radiologist	Quantib
LOCATION				
PZ	51/65 (78.5%)	67/67 (100%)	51/55 (92.7%)	67/72 (93.1%)
TZ	30/39 (76.9%)	42/42 (100%)	30/41 (73.2%)	42/49 (85.7%)
Gleason score				
ISUP 1	23/39 (59%)	36/40 (90%)		
ISUP 2	25/37 (67.6%)	32/37 (86.5%)		
ISUP 3	21/24 (87.5%)	26/26 (100%)		
ISUP 4	9/10 (90%)	12/12 (100%)		
ISUP 5	3/3 (100%)	3/3 (100%)		
PIRADS				
PIRADS 3			10/17 (58.8%)	1/1 (100%)
PIRADS 4			48/54 (88.9%)	56/65 (86.2%)
PIRADS 5			23/25 (92%)	52/55 (94.5%)

## Data Availability

Not applicable.
